# Recent advances in monolithic-integrated lead-based optoelectronic devices

**DOI:** 10.1007/s12200-025-00158-2

**Published:** 2025-06-11

**Authors:** Shaoheng Xu, Jiajun Luo, Haisheng Song, Jiang Tang

**Affiliations:** Wuhan National Laboratory for Optoelectronics, School of Optical and Electronic Information, Huazhong University of Science and Technology, Wuhan, 430074 China

**Keywords:** Monolithic integration, Lead-based optoelectronic devices, Infrared image sensor, Active-matrix display

## Abstract

**Abstract:**

Optoelectronic devices, including light sensors and light-emitting diodes, are indispensable for our daily lives. Lead-based optoelectronic materials, including colloidal quantum dots and lead-halide perovskites, have emerged as promising candidates for the next-generation optoelectronic devices. This is primarily attributed to their tailorable optoelectronic properties, industrialization-compatible manufacturing techniques, seamless integration with silicon technology and excellent device performance. In this perspective, we review recent advancements in lead-based optoelectronic devices, specifically focusing on photodetectors and active displays. By discussing the current challenges and limitations of lead-based optoelectronics, we find the exciting potential of on-chip, in-situ fabrication methods for realizing high-performance optoelectronic systems.

**Graphical Abstract:**

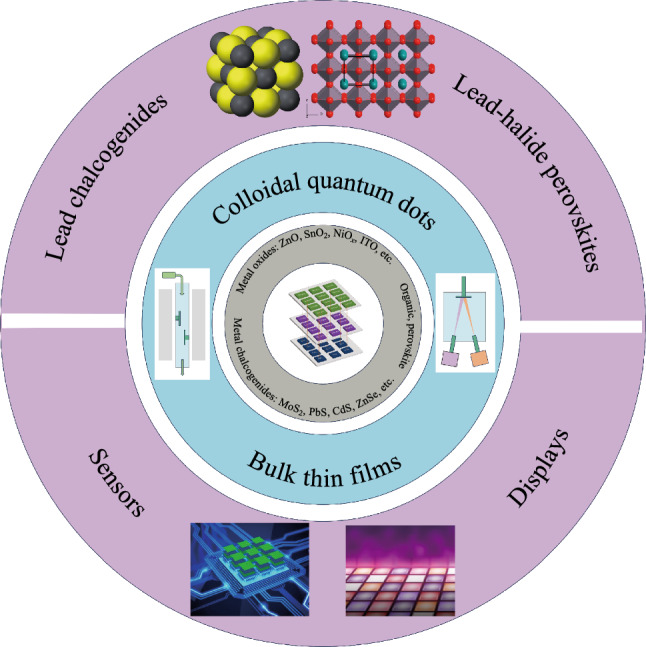

## Introduction

**Fig. 1:**
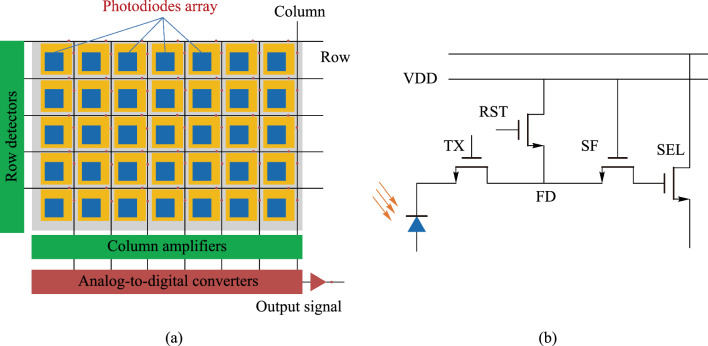
**a** Device architecture and **b** readout circuits schematic of complementary metal-oxide semiconductor image sensors. VDD, the power supply voltage; TX, transfer transistor; RST, reset transistor; FD, floating diffusion; SF, source follower; SEL, row selection transistor

The substantially rising demands for high-bandwidth communication and multifunctional integrated circuits necessitate the integration of optoelectronic devices, such as light sources and photodetectors with silicon-based electronics. This integration, known as optoelectronic integrated circuits (OEICs), offers significant advantages such as high bandwidth, great functionality density, and low-cost production through leveraging mature silicon technology [1–4]. The image sensors and active displays are integrated products of photodiodes and readout circuits. In a complementary metal-oxide semiconductor (CMOS) image sensor (Fig. 1), the two-dimensional photodiodes array converts the incident light into charge carriers, which are stored in a capacitor and subsequently read under the control of readout circuits. After being transformed into digital signals, the image data can be processed and eventually be reconstructed by display devices. The active displays work in an opposite way, where the charge carriers are injected into the diodes and recombine to produce light under the drive of circuits.

Conventionally, integrated optoelectronics has been realized through heterogeneous integration techniques such as flip-chip bonding [5–7]. The III–V compound semiconductor devices are commonly fabricated through molecular beam epitaxy (MBE) or metal–organic chemical vapor deposition (MOCVD) processes at over 500 °C. Such a high processing temperature would cause low-*k* dielectric damage, copper wire disconnection, and impurity diffusion [8]. Therefore, the electric reliability and performance of CMOS devices are degraded. To address these issues, the flip-chip bonding process is proposed, which enables III–V devices and CMOS devices to be fabricated separately. By placing the pixelated device downwards and connecting every pixel to the pixelated circuits through pre-fabricated conductive bumps, the III-V semiconductor optoelectronic devices can be electronically coupled to silicon circuits. This technique offers the advantages of design flexibility, functional expandability, and high interconnect density, making it a mainstream technology. However, significant challenges, e.g., alignment accuracy, hybridization yield, and wafer bowing, arise as bonding pitches scale down to sub-micron levels and wafer sizes increase to twelve inches [9, 10]. In addition, in the heterogeneous integration of micro-LED displays, the prevailing mass transfer printing technique can cause defects and degrade the device’s performance [7, 11, 12]. Therefore, new integration technologies need to be explored to overcome the bottleneck of heterogeneous integration.

To overcome these limitations, monolithic integration, where optoelectronic devices are fabricated directly on the silicon circuits, has emerged as a promising approach. The emergence of lead-based materials, such as lead sulfide colloidal quantum dots (PbS CQDs) and lead-halide perovskites (LHPs), provides a compelling pathway for monolithic OEICs [13]. Lead sulfide (PbS) is a typical narrow bandgap semiconductor and intrinsically sensitive to light with wavelengths up to 2.95 μm at 300 K [14]. The low Auger recombination coefficient, high absorption coefficient, long carrier lifetime, and simple deposition technique make PbS the dominant product for room-temperature and low-cost infrared applications [15–18]. Furthermore, with a large Bohr exciton radius, the bandgap and response band of PbS can be tuned by controlling the size of nanocrystals, which are also called quantum dots. Therefore, the tunable bandgap and simple synthesis render PbS very attractive for full short-wavelength infrared detection [19]. By virtue of those mentioned advantages, the lead-chalcogenide CQDs have become the focus for infrared image sensors and demonstrated near-perfect control over size and shape, resulting in tailorable optoelectronic properties [20–24]. The lead-halide perovskite materials are a group of ionic compounds and have the general formula APbX_3_, where A represents an organic or inorganic cation occupying the cavity between corner-sharing [PbX_6_] octahedra and X represents a halide anion in corner site of [PbX_6_] octahedra [25–27]. This distinctive crystal structure leads to intrinsic softness and dynamic disorder, exhibiting the charming defect tolerance property that is among the foremost concerns for optoelectronic devices [26]. In addition, makes it possible for them to form easily and synthesize nanometer-scale confined crystals under low temperatures. Therefore, the excellent properties make lead-halide perovskites fully exploited in sensors, displays, and photovoltaics.

Low-temperature processing that is compatible with existing silicon technologies is the distinctive feature of lead-based materials. This feature is a major enabling factor for monolithic OEICs. Notably, the devices also exhibit excellent performance. For instance, the specific detectivity of PbS CQDs photodetectors has become comparable to the traditional In_*x*_Ga_1−*x*_As photodetectors operating at uncooled temperatures [23, 28–30]. To date, the PbS CQDs image sensor has been the only technology challenging the III–V counterparts in the same wavelength range. It has successfully entered the stage of large-scale mass production, demonstrating the significant potential of lead-based monolithic OEICs. The disruptive success of PbS CQD image sensors is attributed to the inherent compatibility of CQDs device fabrication with the back-end-of-line (BEOL) processes of CMOS technology.

Recently, the monolithic integration of these lead-based optoelectronics with silicon-based circuits has made significant strides. Here, we summarize the latest progress of lead-based optoelectronic devices for sensor and active-matrix display applications from the perspective of monolithic integration. We will examine this topic from both material fabrication and device integration aspects, and specifically highlight the potential of on-chip integration of these prominent optoelectronic devices.

## Monolithic-integrated lead sulfide based infrared photodetectors

The PbS CQDs imagers have advantages of solution-process and spectrum-tunability. For commercial applications, fast and sensitive imagers need to be explored. Single-element photodetector devices can be fabricated into three architectures: photoconductor, photodiode, and phototransistor [13]. Both photoconductor and phototransistor devices enjoy the gain, which makes them highly sensitive. However, they suffer from difficulty in integration with CMOS circuits thus ruling out their employment for imagers so far. The photodiode device works on the built-in electric field. The photogenerated carriers can be separated swiftly once they diffuse into the depletion region. The temporal response is primarily determined by the diffusion time in the neutral region and drift time across the junction. It has low dark current due to the existence of depletion region but has no gain in principle. Therefore, the photodiode structure exhibits low noise and fast response. Furthermore, the vertically aligned structure of photodiode enables its facile integration with read-out circuits.

**Fig. 2:**
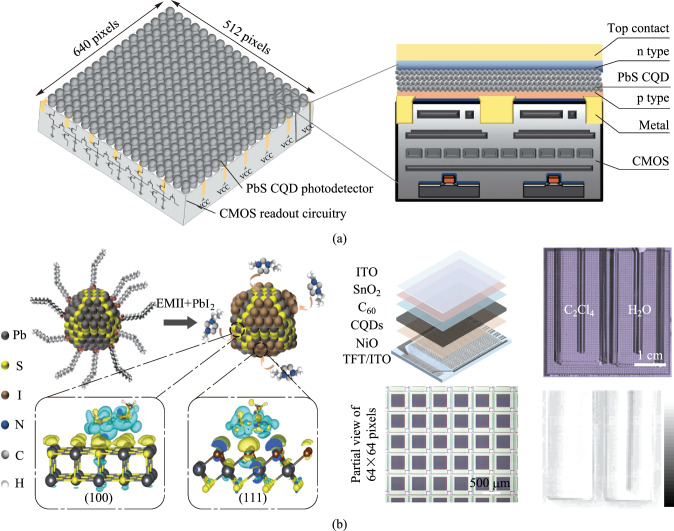
**a** Configuration of a monolithic-integrated PbS CQDs CMOS image sensor with a format of 640 × 512 [28]. **b** Adsorption of EMI^+^ and halide ions on PbS CQDs surfaces and the 64 × 64 TFT imager with 0.35 − 1.8 µm broadband response made by the optimized PbS CQDs [37]. Part **a** reprinted from Ref. [28], Springer Nature Limited. CC BY 4.0. Part **b** reprinted with permission from Ref. [37], Wiley

The architecture of PbS CQDs imager is shown in Fig. 2a, which comprises the vertically coupled PbS photodiodes and CMOS readout circuits. The creation of absorption layer of CQDs image sensor involves three independent processes: synthesis of CQDs, ligand exchange to prepare CQD ink, and deposition of the CQDs film on readout circuits [18, 31]. Then, the charge extraction layer and electrode layer are deposited successively. Lead sulfide CQDs are usually synthesized by hot injection or cation exchange methods with the first exciton absorption being adjustable up to 2600 nm. Through injecting the organic ligands at a specific time to cap the growing CQDs surface, the designated CQDs size and shape with desirable physical and chemical properties can be achieved [32]. The subsequent ligand exchange process modifies the surface ligands to minimize the distance between CQDs when processed into solid films while still enabling stable dispersion of CQDs in solution. Finally, the CQDs self-assemble as a solid film onto the readout circuit surface via a variety of methods, such as spin-coating, spray-coating, ink-jet printing [13, 19, 33]. The image sensor performance depends on the quality of CQDs material and solid film, and on the device performance, which can be optimized by precursor engineering, ligand engineering, and device engineering [34].

A critical challenge for fast and sensitive PbS CQDs image sensors arises from the presence of trap states in the absorption layer and the hybrid interface of photodiodes. These traps can capture the flowing photogenerated carriers, either releasing to the conduction band or recombining with opposite-charged carriers [35, 36]. This process significantly slows the response and decreases the efficiency of photogenerated carriers being collected by electrodes, thereby posing a huge negative impact on the sensitivity and temporal response. Hence, passivating the trap states within the CQDs film and at the device interfaces is crucial. In 2022, Liu et al. demonstrated a monolithic-integrated PbS CQDs CMOS image sensor with a high external quantum efficiency of over 60% operating at 970 nm by effectively minimizing interface states [28]. The reported photodiode device has a configuration of NiO_*x*_/PbS CQDs/C_60_/ZnO (Fig. 2a), in which the NiO_*x*_ and ZnO charge extraction layers were deposited using sputtering, the PbS CQDs layer was spin-coated, and the C_60_ interfacial layer was thermally-evaporated. The three processes feature low-thermal-budget and BEOL-compatibility, avoiding the degradation of CQDs layer quality and the production of high-density interface states. After the introduction of the C_60_ layer, the interface states density decreased by more than half to 2.3 × 10^16^ cm^−3^. Optimization of the CQDs film thickness to 600 nm balanced dark current and external quantum efficiency, resulting in the top-illuminated photodiodes with an external quantum efficiency of as high as 63% at 970 nm (−0.5 V bias), a transient rising time of 0.49 μs, and a − 3 dB bandwidth of 140 kHz. For the PbS CQDs CMOS image sensor, the pixel size and pixel pitch are defined by the bottom pixelated electrode because of the small lateral carrier diffusion length. The 640 × 512 format imager demonstrated a high resolution of 40 lp/mm, even higher than that of In_*x*_Ga_1−*x*_As imager.

The ligand engineering is also frequently used to improve the temporal response of PbS CQDs photodiodes. In 2024, Liu et al. reported a monolithically integrated PbS CQDs TFT responsive up to 1.8 μm [37]. Unlike the usage of solely PbX_2_ (X = I or Br), in which the halide anions are prone to combine with (111) surface, a planar 1-ethyl-3-methylimidazolium (EMI^+^) cation was added as the second ligand, which adsorbed on (100) facets with a face-to-face configuration and is more stable to bind on (100) facets than halide anions (Fig. 2b). The large planar ligand prevented the fusion of CQDs and enhanced the size uniformity, giving rise to the photoluminescence lifetime was doubled. Therefore, the low dark current density of 14 nA/cm^2^ and a high peak external quantum efficiency of 62% at 1.65 µm were achieved. The integration with a-Si TFT readout circuit was facile and was demonstrated to detect the objects behind a silicon wafer and discriminate the colorless tetrachloroethylene and water, which could not be identified by human eyes. Improving the ratio of bromine-to-iodine dual ligands can also lead to well-passivated CQDs [35]. The usage of a second ligand to passivate large-size PbS CQDs is necessary because a larger fraction of nonpolar (100) surface exposed, which is challenging to be passivated by single halide ion and thus more susceptible to form traps on the surface due to oxygen and water erosion [38].

The other aspect influencing the temporal response is the film thickness of absorption layer, which poses a trade-off with quantum efficiency. Reducing the film thickness can decrease the diffusion time of photogenerated carriers, improving response speed, while simultaneously causing optical loss, leading to lower quantum efficiency. Incorporating the BEOL-compatible optical structure, such as optical resonance, can enhance the photon utilization, compensating the drawback of thinner film. The first optical resonance engineering used in PbS CQDs devices was proposed by Ouellette et al. in 2016 [39]. They employed SiN_*x*_ and SiO_2_ layer deposited by plasma-enhanced chemical vapor deposition (PECVD) at 390 °C, which may cause damage to the CMOS readout circuits. Actually, at some specific thickness of the PbS CQDs layer, the Fabry–Perot cavity can be in-situ formed between ITO and Au electrode and the cavity width can be adjusted with the variation of film thickness. This was demonstrated by the research of Deng et al*.* [36]. The absorptance of 50 nm PbS CQDs film at 1330 nm increased by 2.5 times. With a 100 nm PbS CQDs absorbing layer, a record fast response time of 4 ns and a high external quantum efficiency of 42% at 1310 nm was achieved. The in-situ resonant cavity strategy can also potentially be used in other type of photodetectors, such as photoconductor-based devices, i.e., planar bilayer heterojunction, trapping-mode devices [19, 40]. This strategy provides a feasible way for monolithic integration of high-sensitive wavelength-selective response imager.

**Fig. 3:**
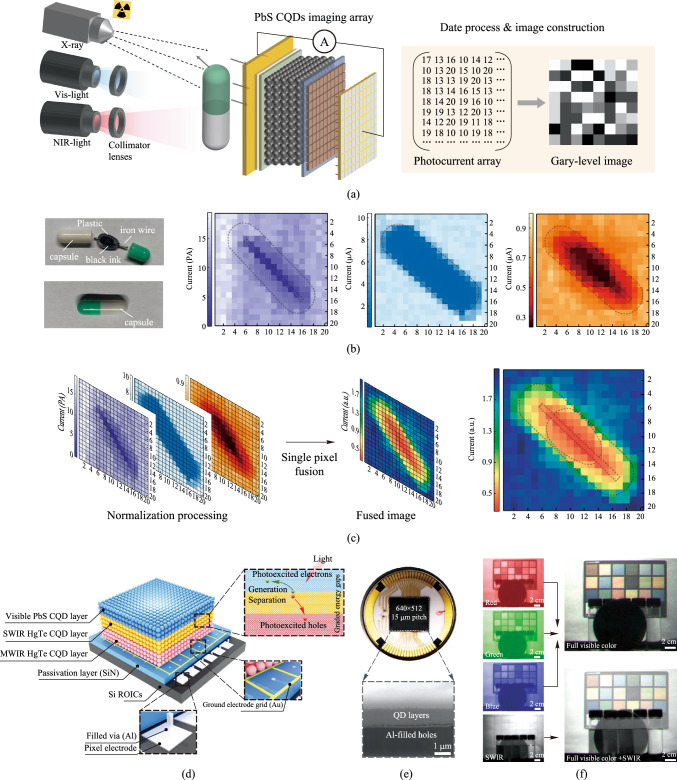
Broadband colloidal quantum dots image sensor response. **a** Schematic illustration of PbS CQDs photodiode array X-ray, visible and infrared imaging. **b** Photograph of a capsule inside and outside captured by a smart phone silicon imager and images of the capsule obtained by the photodiode array under X-ray (purple), visible (blue) and NIR (red) illumination. **c** Date process for pixel-level image fusion and fused image of X-ray, visible and NIR images [29]. **d** Schematic of the ultrabroadband PbS/HgTe CQDs imager. **e** Photograph and cross-sectional SEM image of the ultrabroadband imager. **f** Multispectral imaging mode of the ultrabroadband imager using red, green, blue and SWIR optical filters, and the reconstructed full color image [41]. Parts **a − c** reprinted from Ref. [28], CCBY 4.0. Parts **d − f** reprinted from Ref. [29], Springer Nature Limited

Recently, pixel-level broadband image sensors have been reported. Conventionally, the multispectral imaging is realized by multi-spectral image fusion, where the images captured from several different single-band imagers are merged with the help of algorithms. This method suffers from pixel mismatch and a complicated system. With the pixel-level broadband response imager, precise information and simple devices can be achieved. In 2023, Liu et al. reported a flexible and broadband PbS CQDs image sensor (Fig. 3a). This imager was fabricated on a flexible polystyrene naphthalate substrate and had a response range spanning from X-ray to near-infrared. The key advantage of this imager was its ability to perform pixel-level multispectral image fusion, which could provide more information without pixel mismatch and complex imaging processing [29]. In 2024, Mu et al*.* reported an ultrabroadband imager with a response spectrum from visible to mid-wave infrared. The pixel photodiode was made of stacked PbS/mercury telluride (HgTe) CQDs (Fig. 3d). With optimized graded energy gaps, the imager enabled the separation and collection of photoexcited carriers in different CQD layers, resulting in a broadband spectral response from 0.4 to 5.0 µm. The fabricated 640 × 512 focal plane array (FPA) imager had a low photo-response non-uniformity of 6% and a noise equivalent temperature difference as low as 34 mK [41]. These studies open up new possibilities for the field of imaging.

## Lead-chalcogenide bulk thin film infrared photodetectors

Over the past two decades, intensive research has positioned PbS CQDs as one of the most transformative technologies in the field of infrared detection, culminating in the commercialization of monolithically integrated PbS CQDs sensors. In addition, the PbS bulk thin film infrared detectors have also made progress with vapor phase deposition technique. This might be a possible solution for the high-yield large-scale fabrication of PbS photodetectors.

The PbS (Se) polycrystalline thin film photodetectors have recently garnered renewed interest owing to their process versatility, conformality, broad wavelength coverage and room temperature operation. The PbS (Se) bulk thin film is preferably deposited via liquid-phase method, i.e., chemical bath deposition (CBD), which was utilized since the 1930 s and remains widely employed in high-performance commercial PbS photoconductor products [42–44]. The chemical bath deposition of PbS thin film is carried out at room temperature, denoting the potential for monolithic integration. In addition, the versatility and conformality of CBD process enable film fabrication on non-planar surfaces, such as biomimetic electronic devices, which endows the sensor with a wide-angle field of view, low aberrations, high acuity to motion, and a near infinite depth of field [45–48]. However, due to the lack of precisely controlled chemical bath deposition, the large-scale and high-yield production of low defect density PbS (Se) thin film photodetectors is limited [49–51].

In recent years, Qiu et al. explored uncooled PbS (Se) infrared detectors by vapor-phase deposition. The thin films exhibited self-assembled rodlike microstructures, reminiscent of the morphology made by chemical bath deposition [15, 16, 52]. The PbSe and PbS photoconductors exhibited good detectivity of 2.4 × 10^9^ and 1.9 × 10^11^ cm Hz^1/2^/W under 298 K, respectively. However, a significant challenge for the photoconductors arises from the requirement of being sensitized at temperatures as high as 500 °C in oxygen or iodine atmosphere, which is not possible for monolithic integration with silicon-based readout circuits. They also reported PbSe/CdSe heterojunction photodiodes made by the vapor-phase deposition without erosive atmosphere sensitization process, while the deposition temperature of PbSe film was 400 °C and lasted for 2 h. This process could also cause damage to the silicon-based readout circuits.

Despite their great potential of monolithic integration, the current CBD-based PbS photoconductors are needed to be processed under temperature over 400 °C for several hours to achieve a specific detectivity of 10^11^ Jones. The vapor-phase deposition processes are also conducted at high temperature. In addition, the sensitized photoconductor usually has a response time of several hundreds of microseconds and a large dark current density. This hindered the application of PbS (Se) infrared photodetectors. Few studies on monolithic-integrated PbS (Se) photodiodes prepared by liquid-phase deposition can be found. Therefore, the uniform and high-performance bulk PbS (Se) photodiodes have remained elusive. It is necessary to enhance the PbS (Se) film quality grown from liquid or vapour phase at low temperature and design effective photodiodes structures.

## Monolithic-integrated lead-halide perovskites displays

The highly defect-tolerant LHPs are intensively explored for display applications due to their bright and narrow emission, widely tunable emission spectrum, and facile monolithic integration [53–55]. LHPs can be processed by liquid-phase and vapour-phase methods. Both LHP bulk thin films and nanocrystal solid films exhibit prominent electroluminescence performance, which is achieved by crystallization control and device engineering. In addition, the intrinsic soft crystal structure endows them low Young’s moduli and mechanical flexibility, making them have potential applications for flexible, stretchable, and wearable display devices. The past few years have witnessed remarkable advancement of lead-based perovskite in the display field, from the single-element functional device to the integrated proof-of-concept products. In this section, we will discuss the advancement of monolithic integrated lead-halide perovskites displays.

### Lead-halide perovskite nanocrystals

**Fig. 4:**
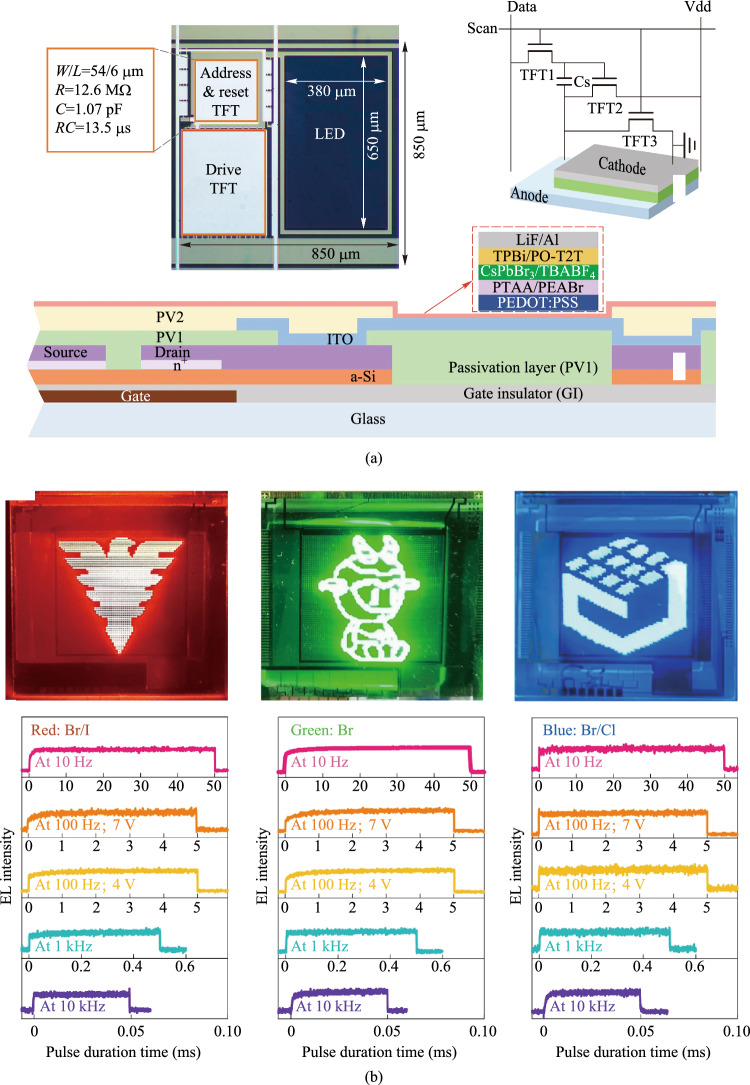
**a** The pixel layout, 3 T1 C pixel circuit, and structure diagram of the active-matrix (AM) TFT display panel with spin-coated perovskite. **b** Digital photographs, electroluminescence (EL) spectra and transient EL intensities of the red, green and blue emissive AM PeLEDs [54]. Parts **a** and **b** reprinted from Ref. [54], CCBY 4.0

The perovskite nanocrystals exhibit narrow-band emission and high photoluminescence quantum yield thanks to the quantum confinement effect [56–62]. This is desirable for achieving high external quantum efficiency and high brightness. However, the PeLEDs usually suffer from ion migration through halide vacancies, which often exist at the surface and grain boundaries. In 2024, Gao et al*.* reported microsecond-response active-matrix perovskite nanocrystal LEDs (AM-PeLEDs) display panels by leveraging the tetrabutylammonium tetrafluoroborate to inhibit the ion migration (Fig. 4) [54]. The display panel was fabricated on a 1.36 cm × 1.36 cm thin-film transistor backplane with a format of 16 × 16 array and a resolution of 30 pixels per inch. This AM PeLEDs display panel exhibits a 5% deviation in both brightness and efficiency uniformity. At a brightness of 1000 cd/m^2^, the peak EQE reaches 23.3%. The half-life of the panel is approximately 10 h at an initial brightness of 100 cd/m^2^. In 2025, Lian et al. reported a localized contact method to prevent non-radiative losses induced by etching process [63]. The PeLEDs made via this process exhibited minimum performance reduction with characteristic pixel lengths from hundreds of micrometres down to about 90 nm. They also fabricated a TFT-powered active-matrix micro-PeLED display with pixel dimensions of 70 μm × 95 μm to demonstrate the display applications.

### Lead-halide perovskite polycrystalline thin films

Considering the success of organic LEDs, vapour phase deposition technique was proposed as an effective route towards the commercialization of PeLEDs [64]. Although the spin-coating-based PeLEDs have been keeping the best performance and has demonstrated various applications, the realization of full-color display was challenged by high-resolution and full-color array patterning strategies. In contrast, vapor-phase deposition methods offer significant advantages. The full-color array patterning can be achieved by fine metal masks and adapted to the existing production lines. In addition, the vapor-phase deposited PeLEDs exhibit the characteristic of monolithic synchronous integration. The perovskite layer, charge transfer layer, and metal electrodes are all in-situ deposited onto the driven circuits. This is a synchronous process, reducing the display panel manufacturing process to just two steps – driven circuit fabrication and device integration.

**Fig. 5:**
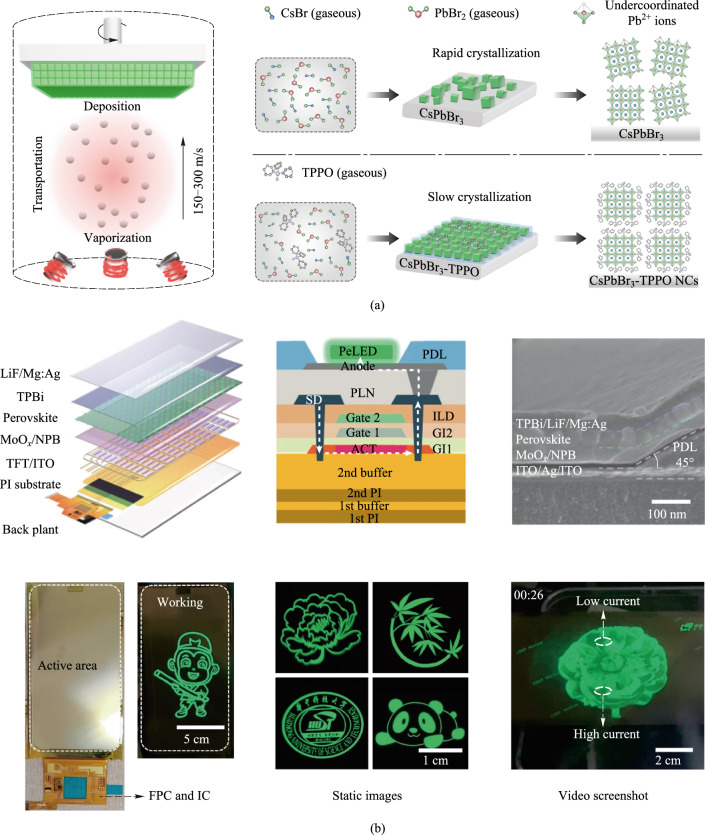
**a** Tri-source thermal co-evaporation and different growth mode of the crystallization process for thermally evaporated CsPbBr_3_ and CsPbBr_3_-triphenylphosphine oxide (TPPO) films. TPPO serves as a surface ligand to simultaneously constrain CsPbBr_3_ nanocrystal growth and passivate surface defects. **b** Device structure and photograph of the CsPbBr_3_-TPPO active-matrix display panel [64]. Parts **a**, **b** reprinted from Ref. [64], Springer Nature Limited

While vapor-phase deposited PeLEDs have historically exhibited lower quantum efficiencies compared to spin-coated devices, primarily attributed to higher defect densities within the perovskite active layer, recent advancements have significantly improved their performance. In 2024, Luo et al. reported an active-matrix perovskite light-emitting diode display panel by directly integrating top-emitting PeLEDs onto a low-temperature polysilicon (LTPS) thin-film transistor (TFT) backplane [64]. The structure is shown in Fig. 5. All functional layers of the PeLEDs, including the hole transport layer, perovskite emissive layer, and electron transport layer, were deposited directly onto the TFT backplane using thermal evaporation under high vacuum conditions. In this approach, the TFT-driven circuits were used as the substrate, and dual precursor sources (CsBr and PbBr_2_) were thermally evaporated as gaseous molecules onto the substrate surface, on which the precursor molecules underwent a chemical reaction, producing CsPbBr_3_ crystal nuclei and then growing to a thin film. The incorporation of a Lewis-base, triphenylphosphine oxide, during deposition played a crucial role in reducing the reactivity of PbBr_2_, constraining the growth of CsPbBr_3_, and passivating the surface states, resulting in well-passivated small CsPbBr_3_ crystal grains. This ensures uniform deposition across the large-area display panel, enabling the display of high-definition images and videos. In addition, the AM PeLEDs featured precise alignment of each pixel with the underlying TFT driver circuit. Finally, the AMPeLED display panel achieved a resolution of 1080 × 2400 and the pixel yield was 98.9% across an area of 6.88 cm × 11.62 cm. During continuous operation, the AM PeLED exhibited negligible brightness degradation.

## Challenges and perspective

While monolithically integrated lead-based optoelectronic devices have demonstrated immense potential for high-performance and cost-effective optoelectronic devices, their practical application must meet the requirements of scale-up fabrication, reliable operation, and low health hazard. Addressing these main obstacles is crucial toward their commercialization.

### Scale-up fabrication

The transition from lab-scale to industrial production faces three fundamental barriers in large-area uniformity, batch-to-batch reproducibility, and process scalability. The spin-coating and vapour-phase deposition have been proven feasible methods for industrial application. However, critical bottlenecks persist in both techniques for CQDs imagers and PeLEDs. In spin-coating process, achieving uniform solvent evaporation and crystallization is challenging, often resulting in high surface roughness and heterogeneous grain sizes [65, 66]. For vapor-phase deposition, controlling the simultaneous deposition of multiple precursors is complex due to varying evaporation rates, which may lead to difficulties in maintaining stoichiometry and film uniformity [67].

Currently, the reported solution-processed CQDs imager exhibit photo-response non-uniformity exceeding 5%, with few below 2% [28, 29, 37, 41]. Moreover, the thickness and performance variations across 8-inch or 12-inch wafer substrates remain unreported, indicating a need for further investigation. For spin-coated and blade-coated active-matrix PeLEDs, many researches have reported uniform deposition over several hundreds of square millimeters with external quantum efficiency non-uniformity above 2% [68–71]. Therefore, achieving more uniform film deposition and device performance remains challenging and needs to be probed.

Although the uniformity issue has not been paid much attention, it is necessary to achieve more uniform films and devices for large-scale fabrication. Based on the fabrication methods, strategies including advanced solvent engineering, additive engineering, and co-evaporation methods could potentially be used to control crystallization dynamics in solution or vapour-phase deposition processes. In 2024, Shi et al. reported the use of binary solvent, including n-octane and n-hexane, to manipulate the fluidic and evaporation dynamics, resulting in balanced Marangoni flow, enhanced ink spreadability, and uniform solute-redistribution [72]. Huang et al. has also summarized the solvent engineering methods for scalable fabrication f high-quality perovskite films in their review paper [73]. However, strategies for controlling the deposition of perovskite films via co-evaporation are rarely reported. In 2024, Dewi et al. reported accelerating the deposition process of co-evaporation MAPbI_3_ by carefully tuning the ratio of PbI_2_ and MAI. They denoted that vacuum evaporation guarantees uniform coatings on large, contoured surfaces and precise thickness control [74].

### Downscaling of pixel size

The pixel size determines the integration density at some extent. Therefore, the downscaling of pixel size is of great concern for CQDs image sensors and active-matrix PeLEDs. The definition of image sensor and active-matrix LEDs is determined by the readout circuits and device size. Due to the low mobility of CQD solid films, crosstalk in CQD image sensors is much lower compared to single-crystalline film imagers. Therefore, image definition is currently defined by the CMOS readout circuits [28]. To enhance integration, optimization of CMOS readout circuits is essential. Similarly, active-matrix display panels are often driven by thin-film transistor (TFT) readout circuits, which commonly have a pixel pitch of hundreds of micrometers. To improve the definition of AM-LEDs, micro-LED technology has been proposed, embracing smaller pixel pitches and higher integration density.

The downscaling of PeLEDs devices can be realized by various methods, including top-down etching, mass transfer, and bottom-up growth. In 2025, Yuan et al. reported the remote epitaxial of perovskite layer with graphene interlayer. Through a transfer process, they fabricated an active-matrix PeLED display with 50 × 50 pixels and a pixel size of 16 μm [75]. Lian et al. reported an effective etching strategy to shirink the size of peroskite active layer. They use a localized contact method to prevent non-radiative losses induced by etching process. This strategy enables nano-scale pixel size. A record LED arrays with a pixel density of 127,000 PPI was achieved [63].

Despite the feasibility of fabricating small size devices, the compatibility of high-temperature epitaxial and deposition processes with silicon-based readout circuits is of concern. Moreover, considering the pixel pitch of readout circuits, which is usually at levels of several micrometers or hundreds of micrometers, the key chellenge for higher definition CQDs imager and AM PeLED displays lies in the pixel pitch of readout circuits, which is determined by the physical limits and fabrication processes of single transistor.

### Reliable operation

High reliability is essential for the long-term operation of optoelectronic devices. The material instability can lead to the degradation of device performance. PbS CQDs are commonly reported to suffer stability issues when exposed to humidity ambient. Under high humidity environment, the surface of PbS CQDs can be hydroxylated, triggering CQDs fusion and leaving inter-band traps [76, 77]. The main instability for lead-based perovskites lies in humidity, oxygen, ultraviolet illumination, ion migration under electric field and thermal degradation [78–82]. For flexible displays and photodetectors, attention should be paid to the structural damage under repeated bending, stretching, or compressing in the device layers [83–85].

From the viewpoint of material quality, strategies including surface and bulk defect passivation are crucial. Recent studies have demonstrated that in situ surface etching and passivation, such as immersing PbS QD films in an I^−^/I_2_ solution, can effectively remove surface Pb oxides and form a PbI_2_ passivation layer, thereby eliminating traps and enhancing film quality and stability [86]. Additionally, introducing ultrathin NiO nanocrystalline interlayers between electrodes and hole-transport layers has been shown to improve hole-extraction efficiency and significantly enhance device stability under thermal and oxidative stress [87]. Halide ion vacancies existing at the surface and grain boundaries are the origin of ion migration [81, 82]. Passivating these halide ion vacancies through additive or interface engineering, such as using monohydrochloride bulk passivator and surface anchoring agent, can eliminate undercharged Pb species and dangling S sites, thereby increasing ion migration activation energy and suppressing the performance degradation [88]. For flexible devices, with low-dimensional materials as active layer, long alkyl chain molecules and polymers as additives, and metal nanowires as electrodes, the mechanical flexibility can be improved [89–91].

The degradation over humidity, oxygen, and light exposure can also be alleviated through encapsulation. As demonstrated in OLED technology, advanced encapsulation schemes such as inert gas, resin filled within glass or metal cover, can achieve water vapor transmission rates below 10^−4^ g/m^2^/day [80]. This provides efficient protection for high-performance devices. Polydimethylsiloxane and aluminum oxide were also used as encapsulation materials. Choi reported that water vapor transmission rate of PDMS/AlO_*x*_ film is 5.1 × 10^−3^ g/(m^2^⋅d) at 45 °C and 65% relative humidity [92]. The devices exposed to 45 °C and 65% RH demonstrated a performance drop less than 5% after 300 h. In 2025, Emery et al. presented a more detailed discussion on tips and tricks for efficient encapsulation in their review paper [93].

### Toxicity

Lead is a hazardous substance, and its widespread use in optoelectronic devices might raise significant environmental and health concerns. The volatile organic solvent used during film fabrication is also of concern. Therefore, developing innovative low-solvent-consumption in situ deposition is of great significance. Besides, preventing the leakage of lead compounds during the device’s whole life cycle also has a significant impact, which can be realized by encapsulation and recovery system [94].

Recent advancements in encapsulation technologies have demonstrated promising results in mitigating lead leakage. For instance, a study introduced a cation-exchange resin (CER) combined with UV resin as an encapsulant, which effectively captures over 90% of Pb^2+^ ions from degraded perovskite solar cells under various environmental conditions, reducing lead leaching to below 5 ppm and meeting the Resource Conservation and Recovery Act (RCRA) standards [95]. Another approach involves the use of hydroxyapatite (HAP) composites decorated with iron, which enhances the adsorption density of Pb^2+^ ions, achieving residual lead concentrations below 15 ppb, aligning with the U.S. Environmental Protection Agency (EPA) standards [96].

In terms of recycling, innovative methods have been developed to recover lead from discarded perovskite solar cells. One such method utilizes ultrasonic water leaching, providing an environmentally friendly and efficient means to extract lead without the use of toxic organic solvents [97]. Recently, Xiao et al. reported the use of sodium acetate, sodium iodide and phosphoric acid to restore near all components from MAPbI_3_ and FAPbI_3_ perovskite [98].

Despite these advancements, challenges remain in ensuring the long-term environmental safety of lead-based optoelectronic devices. Encapsulation materials must be durable against environmental stresses, and recycling processes need to be scalable and cost-effective. Continued research and development in these areas are essential to meet environmental safety requirements and facilitate the sustainable commercialization of lead-based optoelectronic technologies.

On the other hand, considering the current restriction on lead in electronic products is 1,000 ppm, seeking for lead-free optoelectronic materials is still in great need. Very recently, a few lead-free quantum dots detectors and perovskite luminescence materials have been reported. For example, silver telluride CQDs exhibit a sensitive spectra range from 350 to 1600 nm, and lanthanide-based vapour-phase deposited blue emissive materials have achieved photoluminescence quantum yields (PLQYs) of 69% [99, 100].

Ag_2_Te CQDs exhibit sensitivity across a room-temperature detectivity reaching approximately 10^12^ Jones. Additionally, they offer a linear dynamic range exceeding 118 dB and a 3 dB bandwidth over 100 kHz. Importantly, Ag_2_Te CQDs are synthesized using phosphine-free methods, enhancing their environmental compatibility and facilitating integration into consumer electronics [99]. Bismuth-based perovskite quantum dots, such as FA_3_Bi_2_Br_9_, have achieved photoluminescence quantum yields (PLQYs) up to 52% in the blue emission region (~ 437 nm). This high PLQY is attributed to effective surface passivation and strong exciton binding energy, which suppress non-radiative recombination. These materials also demonstrate good air and ethanol stability, making them suitable for light-emitting diode (LED) applications [101].

While lead-free materials have made significant progress, their performance metrics, such as PLQY and stability, are still generally lower than those of lead-based counterparts. However, based on strategies explored in lead-based devices, ongoing research focusing on surface passivation, defect management, and compositional tuning holds promise for narrowing this performance gap.

## Conclusions

Lead-based optoelectronic materials are typical ionic compound semiconductors. These intrinsic material properties render them the possibility of processing under significantly milder conditions, such as low temperatures. Furthermore, the high optical absorption coefficient of lead-based materials, attributed to the contribution of lead atoms to their conduction and valence bands, enables the realization of optoelectronic devices with relatively thin active layers. As a result, lead-based optoelectronic materials make it possible to prepare tailorable high-performance semiconductor optoelectronic devices with low-temperature processes, rendering material synthesis control from zero-dimensional to three-dimensional, photodetection from high-energy particles to mid-infrared light photons, and efficient light-emitting devices.

The compatibility of lead-based materials process with silicon technology is a key advantage. This will enable more efficient optoelectronic integration. At present, integrated III-V devices primarily rely on flip-chip bonding, while spin-coated lead-based devices have demonstrated the cost-effectiveness of monolithic integration. Furthermore, directly growing patterned crystalline or nanocrystal thin films and devices onto the silicon-based circuits by vapour-phase evaporation and liquid-phase deposition can further reduce the complexity of optoelectronic integration and greatly demonstrate the cost and performance advantages. This approach offers another dimension for monolithic integration, where the functional material synthesis and active layer fabrication are synchronous. No pre-synthesis of active materials, i.e., quantum dots, is needed. Therefore, the process complexity is largely reduced and the device performance is improved, which unlocks new possibilities of monolithic integration of lead-based optoelectronic devices. Although the lead-based materials face stability challenges, ongoing research is addressing these concerns through innovative materials engineering and device design. Therefore, the lead-based optoelectronic devices with monolithic synchronous integration characteristics will offer a promising pathway for the future of monolithic optoelectronic integrated circuits.

## Data Availability

The data that support the findings of this study are available from the corresponding author, upon reasonable request.
